# Multiplex immune protein profiling of fine‐needle aspirates from patients with non‐small‐cell lung cancer reveals signatures associated with PD‐L1 expression and tumor stage

**DOI:** 10.1002/1878-0261.12952

**Published:** 2021-05-01

**Authors:** Bo Franzén, Kristina Viktorsson, Caroline Kamali, Eva Darai‐Ramqvist, Vitali Grozman, Vasiliki Arapi, Petra Hååg, Vitaliy O. Kaminskyy, Per Hydbring, Lena Kanter, Sven Nyrén, Simon Ekman, Luigi De Petris, Rolf Lewensohn

**Affiliations:** ^1^ Department of Oncology‐Pathology Karolinska Institutet Stockholm Sweden; ^2^ Theme Cancer, Medical Unit Head and Neck, Lung, and Skin Tumors Thoracic Oncology Center Karolinska University Hospital Stockholm Sweden; ^3^ Department of Clinical Pathology and Cytology Karolinska University Hospital Stockholm Sweden; ^4^ Department of Molecular Medicine and Surgery Karolinska Institutet Stockholm Sweden; ^5^ Department of Radiology Karolinska University Hospital Stockholm Sweden

**Keywords:** biomarkers, fine‐needle aspiration, immune signaling, non‐small‐cell lung cancer, PD‐L1, proximity extension assay

## Abstract

Biomarker signatures identified through minimally invasive procedures already at diagnosis of non‐small‐cell lung cancer (NSCLC) could help to guide treatment with immune checkpoint inhibitors (ICI). Here, we performed multiplex profiling of immune‐related proteins in fine‐needle aspirate (FNA) samples of thoracic lesions from patients with NSCLC to assess PD‐L1 expression and identify related protein signatures. Transthoracic FNA samples from 14 patients were subjected to multiplex antibody‐based profiling by proximity extension assay (PEA). PEA profiling employed protein panels relevant to immune and tumor signaling and was followed by Qlucore^®^ Omics Explorer analysis. All lesions analyzed were NSCLC adenocarcinomas, and PEA profiles could be used to monitor 163 proteins in all but one sample. Multiple key immune signaling components (including CD73, granzyme A, and chemokines CCL3 and CCL23) were identified and expression of several of these proteins (e.g., CCL3 and CCL23) correlated to PD‐L1 expression. We also found EphA2, a marker previously linked to inferior NSCLC prognosis, to correlate to PD‐L1 expression. Our identified protein signatures related to stage included, among others, CXCL10 and IL12RB1. We conclude that transthoracic FNA allows for extensive immune and tumor protein profiling with assessment of putative biomarkers of important for ICI treatment selection in NSCLC.

## Graphical abstract

We analyzed fine‐needle aspirate (FNA) samples of thoracic lesions of non‐small‐cell lung cancer (NSCLC) patients by multiplex protein profiling. Data analysis revealed immune and oncogenic signaling proteins correlated to PD‐L1 expression and tumor stage. Results show that minimally invasive FNA sampling permits profiling of key proteins which may open for longitudinal monitoring to guide immune therapy in NSCLC.

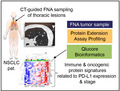

**Abbreviations**
AJCC: American Joint Committee on Cancer staging system
BM: biomarker
CNB: core needle biopsy
CT: computed tomography
FNA: fine‐needle aspiration biopsy
ICC: immunocytochemistry
ICI: immune checkpoint inhibitors
IHC: immunohistochemistry
LOD: limit of detection
NPX: normalized protein expression
NS: NanoString technology
NSCLC: non‐small‐cell lung cancer
PEA: proximity extension assay
RIPA: radioimmunoprecipitation assay buffer
ROSE: Rapid On‐site Evaluation
TMB: tumor mutation burden

**Protein abbreviations**
ADA: adenosine deaminase
ANGPT1: angiopoietin 1 (ANG‐1)
CCL3, CCL4, CCL13, CCL20, CCL23: C‐C motif chemokine ligands 3, 4, 13, 20, and 23
CFS‐1: macrophage colony‐stimulating factor 1
CPE: carboxypeptidase E
CXCL10: CXCL5, C‐X‐C motif chemokines 5 and 10
EGFR: epidermal growth factor receptor
EphA2: ephrin type A receptor 2
FGF2: fibroblast growth factor 2
GZMA: granzyme A
hK8: kallikrein 8 (KLK8)
ICOSLG: ICOS ligand
IL12RB1: IL‐12 receptor subunit beta‐1
IL‐6, IL‐7, IL‐8, IL‐12: interleukins 6, 7, 8, and 12
LAG3: lymphocyte‐activation gene 3
MAD homolog 5: mothers against decapentaplegic homolog 5 (SMAD5)
MCP‐2: monocyte chemoattractant protein 2 (CCL8)
MIC‐A/B: MHC class I polypeptide‐related sequence A and B
MMP12: macrophage metalloelastase
PD‐L1: programmed cell death 1 ligand 1 (CD274)
SPARC: secreted protein acidic and rich in cysteine (osteonectin)
TCL1A: T‐cell leukemia/lymphoma protein 1A
TNFRSF4: tumor necrosis factor receptor superfamily member 4 (OX40L)
VEGFR2: vascular endothelial growth factor receptor 2
WIF‐1: Wnt inhibitory factor 1
XPNPEP2: Xaa‐Pro aminopeptidase 2

## 1 Introduction

Immune checkpoint blockade (ICI) using PD‐L1 or PD‐1 inhibitory antibodies represents a breakthrough for the treatment of non‐small‐cell lung cancer (NSCLC) patients with metastasis where no targetable mutation is revealed [1, 2, 3]. Thus, these antibodies which attack the PD‐L1/PD‐1 axis alone [4, 5] or combined with chemotherapy in first‐line have significantly prolonged median overall survival of metastatic NSCLC patients [6]. As only a fraction of the NSCLC patients will benefit from such treatment and given the risk of potentially severe side effects, there is a need for further patient selection and tumor phenotyping. In the present work, we asked if fine‐needle aspiration biopsies (FNA) which are minimally traumatic can be used for analyzing immune‐ or oncogenic signaling in small thoracic lesions.

Currently, therapeutic decisions for ICI—regimens in stage IV NSCLC are based on immunohistochemistry (IHC) analyses of PD‐L1 expression in surgical specimens or on core needle biopsies (CNB) and in certain cases, with immunocytochemistry (ICC) of FNAs [7, 8, 9, 10, 11, 12]. There has been an extensive search for biomarkers (BMs) that could guide therapy beyond PD‐L1 expression and results have revealed that high tumor mutation burden (TMB), as well as a T‐cell inflamed signature, may further stratify NSCLC patients into responders *vs*. nonresponders to ICIs [13]. Such analyses are though yet not implemented into clinical routine and are hard to use in diagnostics [14].

Thus, to improve the outcomes of first‐line ICI regimens and/or to select optimal second‐line therapy, methods collecting molecular data related to therapeutic response of tumors lesions are required and should include BMs linked to tumor and/or surrounding immune environment [15, 16, 17]. Such methods need to be robust, minimally invasive yet informative and be based on sampling procedures which also capture inter‐ and intratumor heterogeneity. In this sense, FNA‐based tumor and tumor environment sampling combined with molecular profiling may be an opportunity [18]. However, it is a well‐recognized clinical challenge to utilize scarce FNA materials that may be obtained via computed tomography (CT)‐guided sampling for extensive molecular profiling in addition to the current diagnostic routines, for example, mutation analysis and routine immunostainings.

We recently showed that multiplex profiling at both mRNA (by NanoString technology, NS) and protein levels (by proximity extension assay, PEA) can quantify hundreds of molecular markers in FNA samples [19]. Moreover, we demonstrated that such PEA analyses of FNA samples can provide considerable information also on proteins that regulate the tumor‐immune system interplay going beyond PD‐1‐ and PD‐L1 expression [20]. Lately, it was also elegantly shown that genomic characterization of FNA samples from advanced cancer patients may reveal oncogenic drivers to a similar extent as analyses of CNBs [21], thus further illustrating a role for FNA in monitoring precision cancer medicine treatments. The need of such molecular diagnostics is also clear with respect to treatment selection for inoperable stage III and IV NSCLC. Here, we describe the use of PEA profiling for analyses of FNA samples from various stages of NSCLC to reveal protein signatures as related to immune and tumor signaling, tumor stage, and intratumor lesion heterogeneity.

## 2 Materials and methods

### 2.1 NSCLC patient cohort and FNA sampling

In this study, 14 consecutive patients with tumor lesion(s) within the thoracic region and referred to our clinic were included. There were no prespecified selection criteria of patients, all consecutive patients during the study period were included after consent and those that were confirmed to be adenocarcinomas were subjected to protein profiling (Table 1). The Regional Ethical Committee (no. 2005/588‐31‐4; amendments #2008/136‐32; #2016‐2585‐32/1, #2018/1246‐32/1) approved this study and all patients granted the use of their clinical and tumor material for the analyses in an informed written consent. The methodologies conform to the standards set by the Declaration of Helsinki. The collection of biological material was approved by Stockholm Medical Biobank permits and appropriate Material Transfer Agreement (MTA) for sending tumor samples for analysis outside the Karolinska University Hospital area was at hand according to Swedish legislation. The tumor lesions were sampled at the Karolinska University Hospital, Solna, by computer tomography (CT)‐guided percutaneous transthoracic FNA one or two times depending on the lesions found in each individual patient. The primary objective with this procedure was to obtain sufficient material for diagnosis. All FNA samples (Table 1) were collected by an experienced thoracic radiologist using 25‐Gauge needles as part of routine transthoracic CT‐guided sampling for diagnostic purposes. To verify that the sample was representative Rapid On‐site Evaluation (ROSE) was made by means of May–Grünwald–Giemsa staining of smears and direct microscopy [22] and new samples were taken until diagnostic material was obtained. Residual material from the FNA sample was collected, directly frozen at −70 °C, and used in the subsequent protein profiling analyses.

**Table 1:** Clinical and molecular characteristics of the NSCLC adenocarcinoma cohort. The genomic analysis results are given alongside PD‐L1 expression (estimate of % positive tumor cells) examined by IHC or ICC. The ‘Cell types’ column indicates cytology characteristics of stained samples. The images corresponding to cytology preparations and their staining are shown for selected cases in Fig. S3. ND, not determined.

Patient ID	Sex	Age	Smoking status	Tumor stage (8th TNM)	AJCC stage	Genomic alteration	PD‐L1 status (%)	Cell types in FNA sample Estimate of % tumor cells/% macrophages
3A	F	65	Current	T1bN0M0	1A2	ND	65^a^	35/1
6A	M	75	Former	T2aN0M0	1B	BRAF Exon15 (not V600)^d^	Negative^a^	97/2
10A^b^	F	76	Never	T2aN2M0	3A	EGFR Del19^c^	ND	30/ND
11A	F	58	Former	T1cN0M0	1A3	KRAS Exon2^c^, ^d^	Negative^a^	95/5
13A/B	M	84	Former	T1bN0M1a	4A	No mutation^d^	ND	A: 65/5, B: 95/2
14A	M	77	Former	T2aN0M0	1B	ND	ND	Atypical epithelial cells/ND
15A	M	80	Former	T2aN1M0	2B	No mutation^d^	100^a^	< 5/ ND, partial cell fragments
19A	F	71	Current	T2bN2M0	3A	No mutation^d^	Negative^a^	20/2
20A/B	F	71	Never	T2aN1M1c	4B	ALK Variant 3^a^, ^d^	5^a^	98/2
22A/B	F	77	Former	T1aN0M0	1A1	PIK3CA Exon21^d^	40^c^	A: 50‐60/1‐3, B: < 5/ND
23A	F	70	Former	T1aN1M0	2B	KRAS Exon2^c^, ^d^	60^c^	80/5
24A/B	F	79	Former	T1bN0M0	1A2	KRAS Exon2^c^, ^d^	Negative^c^	75/20
26A/B	F	60	Former	T1mIN0M0	1A1	ND	30^a^	35/2
27A/B	M	73	Current	T1bN0M0	1A2	STK11 Exon4^c^, ^d^	ND	85/10

a Determined by IHC analysis of tissue samples.

b Not analyzed by PEA, see Section 3.2.

c Determined by ICC analysis of FNA samples.

d Mutations tested by NGS Oncomine Solid Tumour panel including 22 genes.

### 2.2 Genomic profiling of tumor samples

Genomic profiling which was part of clinical routine was performed on a subset of the cases (Table 1). For *KRAS*, *EGFR*, *PIK3CA*, and *BRAF* mutation status, the 22‐gene Oncomine^®^ Solid Tumor Pane (Ion S5; ThermoFisher Scientific, Uppsala, Sweden) was applied according to the manufacturer's instructions. *ALK* or *ROS1* fusion status was determined by companion IHC according to standard clinical diagnostic methods.

### 2.3 Immunocytochemistry/Immunohistochemistry analyses of PD‐L1 expression

The tumor cell content of samples was estimated by microscope inspection of stained cytology preparations by an experienced cytologist. Cytology smears and/or FFPE tumor sections were also stained for PD‐L1 using the Ventana PDL1 SP263 clone antibody and evaluated according to the manufacturer and current standard diagnostic routines (Roche Diagnostics assay protocol https://diagnostics.roche.com/us/en/products/tests/ventana‐pd‐l1‐_sp263‐assay2.html).

### 2.4 Preparation of tumor material from FNA needles

For the PEA profiling, tumor materials were extracted from needles using 20–40 µL of ice‐cold RIPA buffer (R0278; Sigma‐Aldrich Sweden AB, Stockholm, Sweden) supplemented with protease inhibitors (Protease Inhibitor Cocktail tablet, No. 0469311600, Roche, Sigma‐Aldrich Sweden AB). After lysis, debris was removed by centrifugation (13 000 **g**, 15 min, +4 °C) and protein concentration was determined using Micro BCA™ Protein Assay (Kit No. 23235; ThermoFisher, Uppsala, Sweden). All samples were diluted with RIPA buffer to 1 µg·µL^−1^ prior to the PEA analyses which were carried on the Clinical Biomarker Facility, Science for Life Laboratory, Uppsala University, Uppsala.

### 2.5 Proximity extension assay profiling by Immune Oncology and Oncology II panels

For protein expression profiling, the multiplex antibody‐based PEA with the Multiplex Immune Oncology^®^ and Oncology II^®^ panels was used, respectively. The PEA Immune Oncology panel consists of antibody pairs (allowing for very high specificity and sensitivity) against 92 proteins linked to different aspects of immune‐ and tumor/tumor microenvironment signaling, for example, promotion (*N* = 32), or suppression of tumor immunity (*N* = 37), chemotaxis (*N* = 20), and vascular/tissue remodeling (*N* = 32) (https://www.olink.com/products/immuno‐oncology/). The Oncology II panel includes 92 proteins linked to, for example, cell differentiation (*N* = 42), proliferation (*N* = 43), apoptosis (*N* = 34), angiogenesis (*N* = 20), cellular stress (*N* = 23), and immune response (*N* = 32) (https://www.olink.com/products/oncology/). The assays also contain four internal controls allowing quality control of the reactions. Data were preprocessed according to standard operating procedures at the Clinical Biomarker Facility using Olink Wizard for genex software (MultiD Analyses AB, Gothenburg, Sweden) and manually inspected with normalized protein expression (NPX) values used in the subsequent analyses.

### 2.6 Qlucore bioinformatics analyses for filtering of PEA data

For visualization of similarities of protein expression profiles in FNA from different lesions and from different patient samples as well as to filter out protein signatures from the PEA analysis linked to PD‐L1 or tumor stage, the NPX data were processed by Qlucore^®^ Omics Explorer 3.6 (Qlucore AB, Lund, Sweden). Proteins expressed below limit of detection (LOD, as defined by Olink.com as three times the standard deviation over background, please see https://www.olink.com/question/how‐is‐the‐limit‐of‐detection‐lod‐estimated‐and‐handled/) in < 10% of the samples were excluded from further analysis. Variability in the percentage of proteins detected above LOD was used as an elimination factor (using linear model software in‐built algorithms). The analytical steps were as follows: (a) All proteins, and/or proteins with low variance compared to the maximal variance value, were filtered out. (b) Protein expression profiles of samples were analyzed by rank regression analysis (*P*‐value set to 0.05 or less) to detect tentative protein signatures. The obtained results from (a‐b) were further processed using unsupervised hierarchical clustering and principal component analysis thereby visualizing outliers as well as potential protein signatures via heat maps. To increase the probability to detect tumor microenvironment‐related signatures in the analyses, we used patient age, sex, percent proteins < LOD, and raw protein concentration as elimination factors using the qlucore software (Qlucore AB, Lund, Sweden) in‐built tool box. The PEA NPX values were also plotted for individual markers *per se* or in relation to PD‐L1 expression in different samples using graph pad prism software vers.6 (GraphPad Software, San Diego, CA). The software in‐built linear regression tool was used to correlate protein expression to PEA PD‐L1 expression and to calculate significance levels (Bonferroni‐corrected) in different patient cases.

## 3 Results

### 3.1 The NSCLC clinical cohort, FNA sampling, and mutations

The study outline is presented in Fig. S1, and information on the FNA samples from the 14 NSCLC patients forming the study cohort is shown in Table 1.

The results from the analyses of genomic alterations potentially enabling targeted therapy, for example, *EGFR*, *BRAF*, *KRAS*, and *PIK3CA*, are shown in Table 1. Mutations in *KRAS* exon 2 (patients #11; #23, and #24), *STK11* exon 4 (#27*)*, *BRAF* exon 15 not V600 (#6), *PIK3CA* exon 21 (#22), and EML4‐ALK fusion variant 3 (#20) were found while in patients #13, #15, and #19 no mutations were revealed in the analyzed genes (Table 1). As advanced NSCLC patients without mutations in *EGFR/BRAF* or EML4‐ALK/ROS1 fusions are amenable to single anti‐PD‐1 therapy, that is, pembrolizumab or in combination with chemotherapy, the PD‐L1 expression was also analyzed in a subset of the cases (by IHC: #3, #6, #11, #15; #19, #20, #26, and by ICC: #22, #23; #24) (Table 1). Examination by cytology showed that the tumor cell content varied among the samples ranging from almost 100% (# 20) down to ~ 5% (#15) (Table 1).

### 3.2 PEA immune oncology profiling of FNA samples allows extensive protein marker assessment

**Fig. 1:**
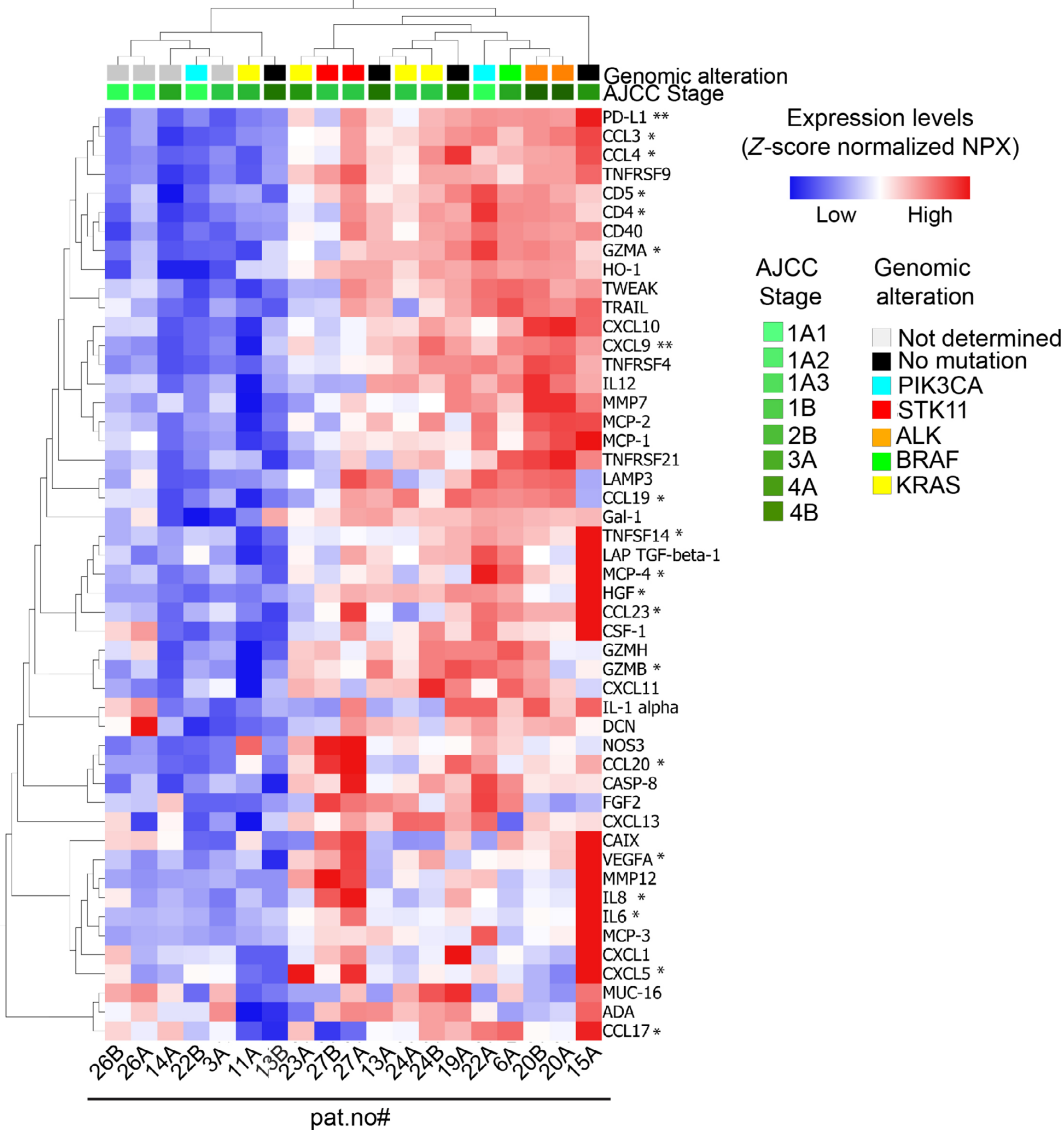
Overview of PEA Immune Oncology data across all NSCLC FNA samples. Heat map of PEA Immune Oncology data organized by unsupervised hierarchical clustering of expression levels (normalized protein expression, NPX) of the top‐49 most variable of the 84 proteins detected (all proteins expressed above LOD in at least 10% of all samples) in FNA samples from NSCLC patients. The sample annotations included are pat.no, detected genomic alteration and refer to data presented in Table 1. Stars indicate biomarker candidates for immune therapy response suggested by Chen *et al*. (*) [23] and Ott *et al*. (**) [13].

FNA samples were profiled for immune and oncogenic signaling proteins using the PEA Immune Oncology panel complemented with PEA Oncology II analyses (Fig. S1). As FNA samples, from which RIPA extracts were obtained for the PEA profiling, were minute in terms of amount, the data generated by PEA analytics were first manually inspected to reveal protein expression coverage across the different samples. The PEA Immune Oncology profiling NPX (Normalized Protein eXpression levels, ^2^log scale) data from the entire sample cohort of all the included 92 protein reactions showed that 84 proteins were detected over LOD (for definition, see Section 2.6) in 18 out of 20 samples. These proteins were used in the further analyses in which data from patient #10 were excluded as the majority of the proteins were under LOD. For an overview of the PEA profiles in individual FNA samples of the NSCLC patients, a heat map organized by unsupervised hierarchical clustering was made and the expression levels of the top‐49 most variable proteins are shown in the context of tumor characteristics, that is, tumor stage, and type of genomic alteration detected (Fig. 1).

As seen FNA samples originating from the same lesion tend to cluster together, that is, pat. #20, #24, #26, and #27. Moreover, functionally related proteins were also clustered adjacent to each other, that is, CCL3/CCL4, IL‐6/IL‐8, and CD4/CD5. Interestingly, 14 of these proteins (marked with star(s)) have previously been associated with acquired ICI resistance [13, 23] pointing toward a potential of PEA to reveal protein signatures in FNA samples which relate to ICI response.

### 3.3 Protein signatures related to immune‐ or oncogenic signaling correlate to PD‐L1 PEA expression levels

**Fig. 2:**
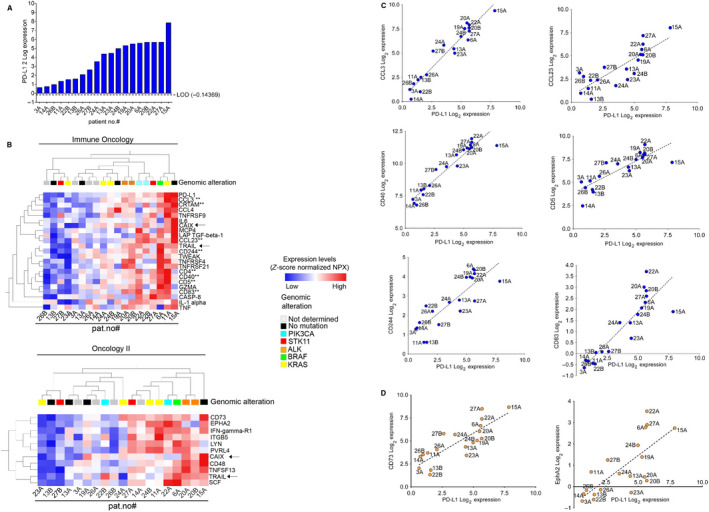
PD‐L1 PEA expression data correlate to protein signatures related to immune or tumor signaling. (A) The PD‐L1 expression as obtained in PEA analyses of the individual patient samples is shown. The dotted line indicates LOD of PD‐L1 in the PEA assay. (B) The PEA data were analyzed by rank regression analyses vs. PD‐L1 expression from the PEA analytics and with LOD data used as elimination factor. Top: Protein markers in PEA Immune Oncology panel which show significance with *P* = 0.05. Markers labeled with ** were also significant with *P* = 0.01. Arrows indicate proteins that were present in the Oncology II panel as well. Bottom: Protein markers in PEA Oncology II data that were significantly correlated to PD‐L1 expression (*P* = 0.01) are shown. Arrows indicate proteins that overlap with the Immune Oncology panel. (C). PD‐L1 from (A) and markers identified in (B, top panel) were plotted across the individual patient samples. All expression values were above LOD, except for CD83 (26% of samples < LOD (−0.24), samples #3A, #11A, #14A, #22B, and #26B). The dotted line indicates linear regression fit using graphpad software. (D) Correlation of PD‐L1 expression to CD73 (left) and EphA2 (right). Expression values from (A) and (B, bottom panel) were used. The dotted line indicates linear regression fit using graphpad software. All correlations were significant (*P* < 0.05) also after Bonferroni correction of *P*‐value for multiple testing. Please note that EphA2 expression in samples #3A, #13B, #14A, #22B, and #23A displayed values below LOD (−0.18).

The PEA Immune Oncology and Oncology II data were next studied in context of PD‐L1 expression as measured by PEA analytics (Fig. 2). Data from oncology II were inspected in the same way as the Immune Oncology data (Section 3.2), which resulted in a total of 163 proteins being analyzed.

PEA monitoring of PD‐L1 expression revealed signals over LOD in all of the NSCLC FNA samples but the magnitude of expression varied > 100‐fold among the different samples (Fig. 2A). In 10 samples, % PD‐L1 positivity of cells was also analyzed *in situ* in either tissue samples (by immunohistochemistry, IHC) or in cytological smears (by immunocytochemistry, ICC) (Table 1). The PD‐L1 assessment by ICC has previously been reported to be valid for assessment [7]. For some cases, immunostaining for PD‐L1 is presented in Fig. S2 and shows the capacity to assess PD‐L1 by ICC or IHC, yet demonstrating heterogeneity in PD‐L1‐positive cells both among samples but also within a sample. The correlation between % PD‐L1‐positive cells (Table 1) and PD‐L1 assessment by PEA analytics (Fig. 2A) revealed a Pearson coefficient of 0.6 which was not significant (data not shown).

Next putative protein signatures related to PD‐L1 expression were analyzed using rank regression on the entire PEA Immune Oncology data set *vs* PD‐L1 (Fig. 2B, top panel). As seen, a 22 protein signature showed correlation (*P* < 0.05, for eight proteins *P* < 0.01) to PD‐L1 PEA expression. Among these were the T‐cell markers CD4, CD5, markers for macrophages, for example, CCL23 as well as proteins expressed by other immune cells, for example, CD244 thus illustrating that the PEA Immune Oncology analytics can capture signals from multiple immune cell types present in an FNA sample. Albeit the number of cases analyzed is limited, several of these individual proteins correlated significantly to PD‐L1 protein expression levels when evaluated by univariate regression analysis (*P* < 0.05, Bonferroni‐corrected *P*‐value) (Fig. 2C). Of note, sample #15A which according to cytology had few intact tumor cells but a high content of mostly degenerative and inflammatory cells including macrophages (which are PD‐L1‐positive) deviated most from the correlation line for multiple markers, for example, CD4, CD5, CD40, and CD83. Sample #15A also showed the highest level of CCL23 and CCL13 (also known as monocyte chemotactic protein 4 (MCP4) which indeed are reported to be expressed in macrophages and/or are linked to their signaling circuit [24]. The rank regression analysis of markers within the PEA Oncology II panel vs PD‐L1 levels revealed an 11 protein signature (Fig. 2B, bottom panel). Among the protein markers was CD73, also known as 5′‐NT and previously reported to act as an immune suppressor in multiple ways within the tumor microenvironment [25, 26, 27, 28]. Another marker was EphA2, an oncogenic receptor tyrosine kinase linked to poor overall survival in NSCLC [29, 30, 31] and involved in EGFR as well as VEGFR2 signaling in NSCLC [32, 33, 34]. Both CD73 and EphA2 also showed a significant correlation to PD‐L1 in univariate regression analysis (Fig. 2D).

Of note, albeit the identified markers correlating to PD‐L1 levels as assessed by the Immune Oncology and Oncology II PEA profiling was statistical significant also after Bonferroni correction *P*‐value for multiple testing, the low number of samples analyzed requires caution when interpreting individual markers. Nevertheless, presented data show that PEA analytics on FNA samples allow for identification of markers in relation to PD‐L1 expression.

To further strengthen the analysis, we implemented a functional network analysis using the String database tool (https://string‐db.org) based on the most significant proteins (*P* = 0.01) of the signatures shown in Fig. 2 (i.e., PD‐L1/CD274, CCL3, CCL23, CD83, CD244, CD40, CD5, CD73/NT5E, and EPHA2). Interestingly, this network analysis (*P* < 1.0e‐16) included automatically also CD48 and TNF, proteins that also were part of the signatures (with less statistical stringency, *P* = 0.05), which would be unlikely if the signatures were just random (Fig. S5).

### 3.4 Immune profiling of NSCLC FNA samples shows association to tumor stage

**Fig. 3:**
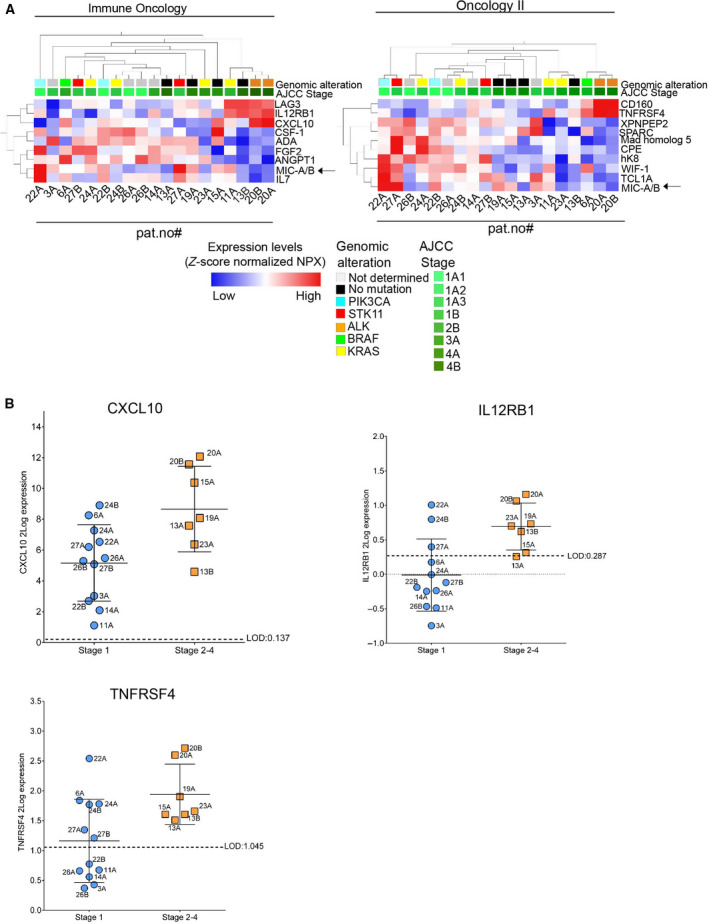
Immune Oncology and Oncology II profiling of FNA samples analyses reveals tentative protein signatures which correlate to tumor stage. (A) PEA data from Immune Oncology or Oncology II data sets were analyzed in relation to tumor stage (Table 1). Rank regression analysis revealed protein signatures that show statistical significant association to stage (*P* < 0.05). Left: PEA Immune Oncology data. Right: PEA Oncology II data. Arrow indicates MIC‐A/B present in both panels. (B) Dot plots of three proteins within the PEA profiles (A) presenting statistical significant (*P* < 0.05, *t*‐test) differences between samples from AJCC stage 1 and stage 2–4, respectively. The LOD value for each marker is indicated by dotted line; the mean value by a solid line and whiskers representing the 25th and 75th percentiles respectively.

As oncogenic drivers as well as infiltration of immune cells may change when a tumor progresses and gets locally invasive or metastatic, it is likely that protein expression profiles are different in tumor lesions of different stages. To examine this, the correlation of PEA Immune Oncology or Oncology II data and clinical stages were analyzed (Fig. 3). For the rank regression presented in Fig. 3, samples from 13 patients with different stages (Table 1) were used with the AJCC stage applied for classification. Out of the 84 markers analyzed within the PEA Immune Oncology data set, a signature of nine proteins showed a statistical significant association to tumor stage (*P* < 0.05), including LAG3, IL12RB1, and CXCL10 (Fig. 3A, left panel). Albeit LAG3 has been suggested as a potential immune checkpoint target, only 3 patient samples in our cohort expressed LAG3 levels above LOD (pat. #11, #13, #20). In contrast, IL12RB1 and CXCL10 were both expressed above LOD in at least half of the studied samples (Fig. 3B). When the same analyses were done using PEA Oncology II profiling data, 10 proteins correlated to tumor stage (*P* < 0.05), for example, CD160, TNFRSF4/OX40L, XPNPEP2, SPARC, MAD homolog 5/SMAD5, CPE, hK8/KLK8, WIF‐1, TCL1A, and MIC‐A/B, (Fig. 3A, right panel). Out of these WIF‐1, TNFRSF4, and CPE were expressed above LOD in a majority of samples. Dot plots representing CXCL10, IL12RB1, and TNFRSF4 show a significant difference between samples from stage 1 and stage 2–4 lesions (Fig. 3B). Here, we also applied the String database tool to examine functional association of proteins which showed correlation to tumor stage. Ten proteins from the signatures in Fig. 3A were identified within the core of the functional network (Fig. S6). This result was regarded significant and further strengthen the relation to tumor stage, although validation is needed. In summary, PEA analytics on FNA samples allow for identification of markers relating to tumor stage yet given the low number of samples in the cohort individual markers should be valued taking this limitation into account.

### 3.5 FNA‐based PEA immune profiling of NSCLC in relation to tumor microenvironment and heterogeneity using PEA‐based analyses

**Fig. 4:**
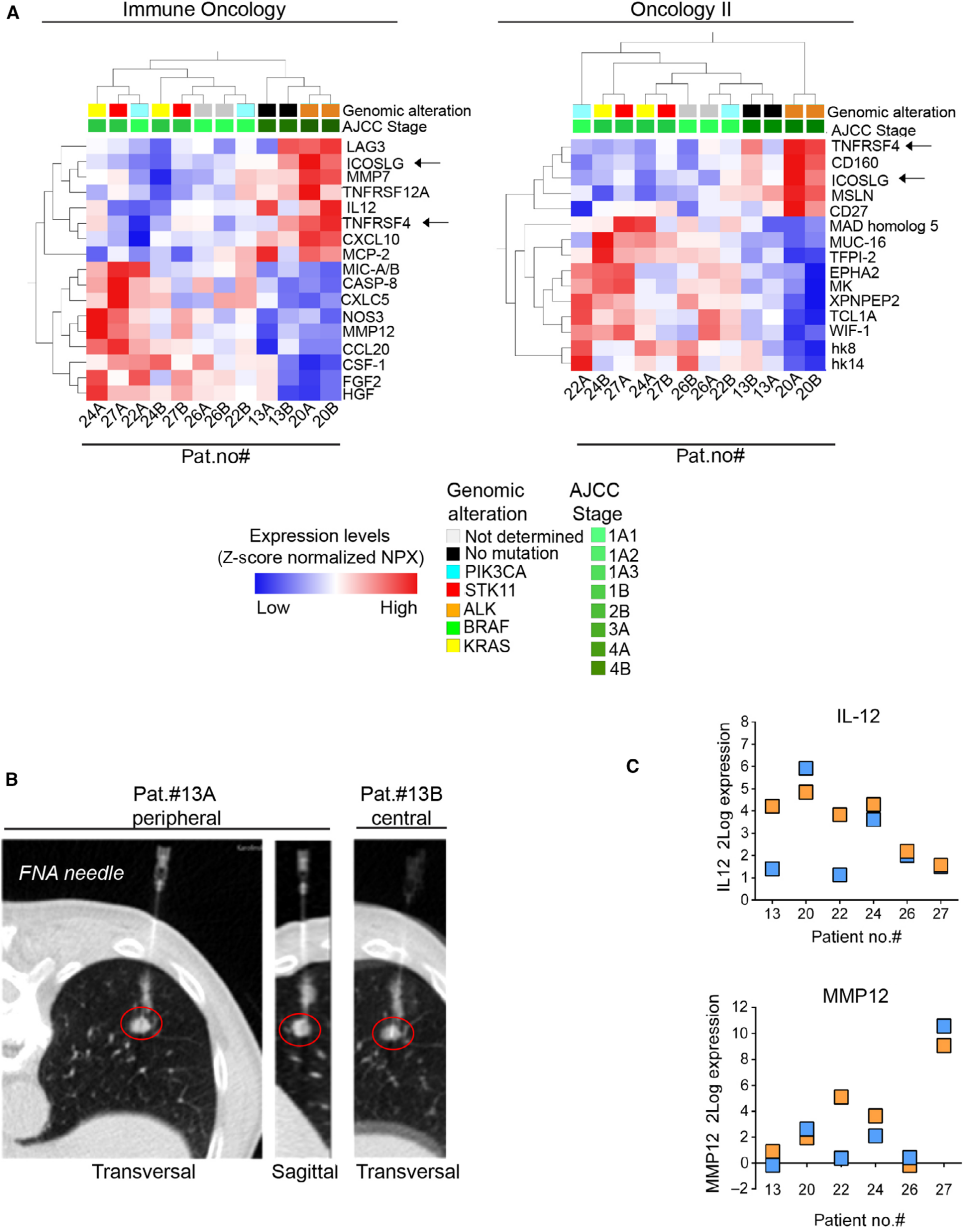
(A, B) PEA profiling of FNA NSCLC tumor samples correlates with tumor cell content. (A) PEA data from Immune Oncology or Oncology II data sets representing pairs of FNA samples from the same tumor lesion in six NSCLC pat. were analyzed in relation to tumor stage (Table 1). Rank regression analysis revealed protein signatures that show a significant association to stage (*P* < 0.05). Left: PEA Immune Oncology data. Right: PEA Oncology II data. Arrows indicate, ICOSLG and TNFRSF4, which were present in both PEA panels. (B) CT images captured during the FNA sampling (the FNA needle can be observed, tumor marked by the red ring) of pat. #13, where sample pat.#13A was obtained from the periphery (confirmed by the sagittal perspective) of the tumor whereas sample pat. #13B was obtained from the central part. Additional CT images from more patients (paired samples only) are shown in Fig. S4. The cytology of these samples is presented in Fig. S3. (C) PEA data from (A) were plotted for IL‐12 and MMP12 across the individual patient sample pairs. Squares represent A (orange) and B (blue) sample pairs.

For establishment of BM signatures in relation to longitudinal monitoring of therapy, it is desirable that the FNA sampling and subsequent protein profiling support the diagnostics by reflecting the heterogeneity seen in the examined lesion. This relates to both tumor‐ and tumor microenvironment‐associated markers. Here, we set out to address this in different FNA samples taken from the same tumor lesion followed by downstream PEA analytics. Thus, in six patients, that is, pat. #13, #20, #22, #24, #26, and #27, two consecutive FNA samples were obtained as part of the clinical routine diagnostic procedure (labeled A/B in Table 1). CT images representing positions of the two different samples from the individual lesions are presented in Fig. S4. The rank regression analyses with respect to tumor stage generated a set of tentative signatures from Immune Oncology and Oncology II panels, respectively (Fig. 4A). The tumor and immune cell content of these six sample pairs (A/B) is shown in Fig. S3 and data given in Table 1.

In FNA samples of the peripheral and the central parts of the tumor from pat. #13 (Fig. 4B, #13A and #13B), higher protein expression levels of, for example, IL‐12 and MCP‐2 were observed in the periphery compared to the central part whereas, for example, LAG3 and ICOSLG showed the opposite pattern. (Fig. 4A, left panel). When FNA samples were analyzed with the PEA Oncology II panel, samples from pat. #13 showed a more homogenous expression pattern in contrast to, for example, #22A and #22B (Fig. 4A, right panel). Nevertheless, cytology showed more tumor cells in sample from pat. #13B *vs* #13A (Fig. S3). Taken together, pat. #13, #20, and #26, all showed relatively similar protein patterns in both samples (A/B) in contrast to #22, #24, and #27 using either of the two PEA panels. These results were in agreement with the cytological assessment of differences between A and B samples (Table 1, Fig. S3).

Two of the markers from the six repeated samples (A and B) shown in Fig. 4A were also analyzed with respect to protein expression levels (Fig. 4C). Results showed that the dynamic range and variability in expression differed between markers, for example, MMP12 which displayed large differences between patients and samples A and B from pat. #22. All in all, albeit sample number is limited, results illustrate that FNA and PEA analytics hold potential to capture tumor heterogeneity in signaling.

## 4 Discussion

The introduction of ICIs has greatly improved the treatment possibilities of disseminated NSCLC tumors [4, 5, 6]. Yet, in clinical settings, inter‐ and intra‐patient heterogeneity of different NSCLC lesions, with respect to ICI response is often seen and calls for longitudinal biomarker (BM) analytics [15, 16, 17]. In this context, sampling of lesions by minimal invasive and atraumatic FNA has emerged as a promising alternative to CNB when combined with ultrasensitive and multiplex molecular profiling methods [18]. For early diagnostics of unknown lesions as well as inoperable tumors of the thorax, FNA should be further explored not only for diagnostics as such but also to retrieve BMs to guide therapy, for example, in the adjuvant setting.

Here, we applied multiplex protein‐based analytics, that is, proximity extension assay (PEA) for profiling of small NSCLC lesions of different stages confined to the thoracic region. PEA has by us and others been shown to hold capacity to reveal protein and/or BM profiles in tumors or plasma from cancer patients [20, 35]. We here for the first time show that PEA can be used to decipher immune and tumor signaling in minute FNA material also from NSCLC lesions. Some of these immune signaling components are reported to be of relevance to response to ICIs [13, 23, 25, 27, 28, 36]. Thus, our study points at a possible path to overcome certain of the challenges for interventional oncology as previously outlined by Schoenberg *et al*. [18]. This means opportunities to obtain biopsies that provide more molecular information at the same time as the sampling for an individual patient may take place longitudinally, that is, to be repeated when new information is needed and still be minimally invasive. Although our study is based on a limited set of samples and results at a detailed level should be treated with caution, we observed that a surprisingly high number of proteins linked to tumor or immune signaling and also reported to be linked to ICI responsiveness were detected above LOD in a majority of these minute FNA samples. In support for data validity and in line with our previous study [20], we observed that FNA samples originating from the same lesions and functionally related proteins tend to cluster together based with regard to their protein profiles.

Interestingly, we identified tentative protein signatures which showed a strong correlation to PD‐L1 PEA expression including CCL3, CCL23, CD4, CD5, CD40, CD73, and CD244. Furthermore, we also observed functional associations between PD‐L1 and multiple of the markers within signatures (Figs S5 and S6). Thus, our PEA analytics could in the future when validated in a larger cohort potentially complement PD‐L1 measurements by ICC which already have been described feasible in NSCLC FNA samples [7, 8, 9, 10, 11, 12]. It is interesting to note that PD‐L1 was detected above LOD (7.6 pg·mL^−1^) in all FNA samples, including samples that were negative for PD‐L1 by ICC or IHC assessment, confirming high sensitivity of PEA technology and a possible future place in tumor diagnostics. Thus, the high dynamic range of PD‐L1 level detection by PEA may provide more robust assessments of PD‐L1 in FNA samples than presently can be achieved by ICC or IHC.

An FNA sample should be regarded as a sample from the tumor and its microenvironment. Given this, we expected to detect protein signatures that reflected not only the tumor cells *per se*, but also various immune cells, for example, regulatory T cells (Tregs), CD4‐positive or CD8‐positive T cells, macrophages of different activation states, NK and dendritic cells. Indeed, about half of the proteins captured within our tentative signature related to PD‐L1 expression have previously been described at gene expression level in relation to different immune cell populations using CIBERSORT [24]. Assuming that such CIBERSORT data also may be applied when studying protein levels, our data suggest higher levels of PD‐L1 to correlate to increased levels of, for example, T cells (CD8 cells, CD4 memory cells, Tregs), NK cells (of different functional classes) as well as M0, M1, and M2 macrophage subtypes (Table S1).

Interestingly, several of the markers of the protein signature that related to PD‐L1 PEA expression levels have earlier been reported to be associated with ICI pembrolizumab response in melanoma based on their mRNA expression pattern, for example, CCL23, CCL13/MCP‐2, CCL4, CD4, CXCL5, CCL20, and GZMA [23], thus further strengthening the potential for FNA and PEA analytics for capturing BMs of relevance to ICI. Moreover, it was shown that a T‐cell inflamed signature on mRNA expression level can be linked to pembrolizumab responsiveness in NSCLC [13]. Indeed, we found that two markers, LAG3 and CD27, which both were expressed at a higher level in more advanced stage tumors, that is, pat. #13 and pat. #20, were overlapping with the T‐cell inflamed signature. In our limited cohort, eight patients underwent surgery and eight patients received combinations of chemo‐, radio‐, and/or targeted therapy. Interestingly, one patient, pat.#15 which demonstrated 100% PD‐L1 positivity in NSCLC tumor tissue by IHC (Table 1) and whose FNA sample showed the highest level of PD‐L1 in PEA analyses was treated with pembrolizumab during several courses and responded well. Thus, a path forward for FNA and PEA analytics may with respect to immune‐ and oncogenic signaling analyses be assessment of samples from a larger cohort of NSCLC patients who in a metastatic setting have been given uniform PD‐1/PD‐L1‐based immune therapy.

Our study revealed a protein signature within Oncology II that correlated with PD‐L1 PEA expression among them were CD73 and EphA2. We and others earlier reported that the EphA2 expression is linked to poor outcome in NSCLC and more recently both EGFR as well as VEGFR2 signaling has been associated with EphA2 [29, 30, 31, 32, 33, 34]. The positive correlation between high PD‐L1 and EphA2 in our study may indicate that EphA2 expressing tumor parts are immune‐suppressed but further analyses are required to prove such a statement given the small sample cohort analyzed. We observed CD73 expression in some of the NSCLC FNA samples but at this point the functional significance of this is unclear. Yet the detection of CD73 is interesting given that CD73 previously has been reported to be immune suppressive in NSCLC and/or other tumor types [25, 26, 27, 28]. Thus, further analyses of CD73 expression in relation to various immune cells in the FNA samples are of high interest.

In our work, we also identified a nine‐protein signature which was associated with tumor stage. Thus, LAG3, IL12RB1, and CXCL10 showed a tendency to have increased expression levels in advanced stages while CFS‐1, ADA, FGF2, ANGPT1, MIC‐A/B, and IL‐7 showed the opposite trend. However, LAG3 and IL‐7 should be treated with caution due to expression below LOD in a large proportion of the analyzed samples. Taken together with the signatures identified when looking on intrasample cellular heterogeneity and according to CIBERSORT data (Table S1), our results may suggest that the T‐cell proportion is higher in advanced stages *vs* early stages together with activated dendritic cells, paralleled with lower relative levels of NK cells [24]. As the sample size of our study is small, further studies are needed to validate such a conclusion.

The observed differences in protein signatures between pairs of samples from the same lesion may be linked to differences in the microenvironment as well as to tumor heterogeneity, which in turn may be reflected by differences in cell composition of the analyzed FNA sample. For example, sample #13A (peripheral, low tumor cell content) and #13B (central, high tumor cell content) showed a relatively similar profile although the expression of, for example, LAG3 and TNFRSF4 (OX40L) was higher in the central parts and, for example, MCP‐2 (CCL8) and IL‐12 showed the opposite expression pattern. This may reflect more macrophages and inflammatory cells in the periphery of the tumor. In contrast, sample #22A and sample #22B were according to the hierarchical clustering more different. Sample #22A also showed a high expression of IL‐12 and CXCL10 (which links to macrophages and dendritic cells according to CIBERSORT data (Table S1) relative to sample #22B. This observation is supported by the cytology that shows high tumor cell content in #22A but less tumor cells mixed with blood in #22B (Fig. 4A, Fig. S3, Table 1).

Signaling in tumor cells *per se* or its microenvironment is an important driver for both intrinsic and acquired therapy resistance to both targeted therapy as well as to ICIs [13, 23, 25, 27, 28, 36]. Here, we report analysis of pairwise consecutive FNA samples from six patients and compared expression of > 160 different proteins. Although most of the tumor lesions sampled were small, results indicate that this approach is feasible. Moreover, our results illustrate that this analytical path has a potential to reflect microenvironment and phenotypic heterogeneity in individual tumor lesions in parallel with levels of specific proteins, which together may in the future be used for understanding ICI response and/or resistance. Yet a prerequisite is that each of the FNA samples has been inspected by a cytologist and verified as representative of the tumor microenvironment as illustrated by our results.

We also want to stress some of the limitations of our study. First, we analyzed a low number of FNA samples which reduces the power and hence the reliability of the statistical calculations. Another limitation is that the heterogeneity of the same lesion was examined on an even smaller number of samples making it difficult to draw in‐depth conclusions albeit it shows that FNA and PEA analytics may assess heterogeneity. Moreover, although PD‐L1 assessment is possible with both IHC on tissue and by ICC [7], the values obtain are difficult to compare and also tough to link to PD‐L1 expression as measured by PEA. From a biological point of view, our study has a limitation in that the PEA on the Multiplex Immune Oncology and Oncology II panels measure only total protein. Thus, we cannot capture changes in protein activity, for example, phosphorylation. For example, it has been shown that although EphA2 total protein expression holds prognostic capacity in NSCLC, EphA2 is also regulated by multiple phosphorylations which impact on its oncogenic function (reviewed in [37]). A further path ahead for our findings is to validate the protein signature obtained *in situ* to assess which cells that express the identified protein signatures. Importantly, all this additional information does not affect the findings of the current study but emphasize the importance of future studies, validating the feasibility of FNA sampling in relation to a defined treatment, for example, ICI, taking a broader profiling into place and which also include *in situ* validation.

In summary, although we in this study used a limited sample set, our results encourage both extended diagnostics using PEA analytics and as related to discovery of factors for therapy decisions at tumor presentation. Moreover, as atraumatic FNA sampling allows for multiple sampling of one or several lesions intended for broad molecular analyses, it could also represent a tool to assess therapeutic targets in the primary tumor as well as metastases longitudinally during the disease course.

## 5 Conclusions

Immune checkpoint inhibitors have a central place in the treatment of NSCLC patients whose tumor lack targetable mutations. Our work addresses an important clinical challenge; that is, how broad molecular diagnostic information from NSCLC lesions can be achieved without extensive traumatic biopsy sampling. Image‐guided transthoracic fine‐needle aspiration (FNA) sampling offers several advantages for the patient but provides on a routine basis only scarce materials for cytology, immunocytochemistry, or other diagnostic protein‐based biomarker analyses. The present work represents a proof of concept for the successful application of multiplex and ultrasensitive molecular assessments of protein signatures and key immune targets, for example, PD‐L1, using minimal residual FNA materials of NSCLC lesions. Thereby, additional traumatic sampling may be avoided. Our proposed procedure also paves the way for and facilitates longitudinal extensive molecular monitoring of both primary and metastatic lesions before and during immunotherapy as support for clinical therapeutic decisions.

## Conflict of interest

The authors declare no conflict of interest.

## Author contributions

BF, KV, CK, LK, SE, LDP, and RL conceived and designed the project. BF, KV, CK, ED‐R, VG, SN, SE, and LDP acquired data. BF, KV, CK, ED‐R, VG, SN, LDP, SE, and RL analyzed data. All authors contributed to data interpretation. BF, KV, CK, and RL wrote the original draft and all authors reviewed and edited the draft. KV, RL, BF, SE, LDP, and PHy acquired funding support for the study.

### Peer Review

The peer review history for this article is available at https://publons.com/publon/10.1002/1878‐0261.12952.

## Supporting information


**Fig. S1**. Overview of the study.
**Fig. S2**. Examples of (A) immunocytochemistry (ICC) and (B) immunohistochemistry (IHC) analyses of PD‐L1 expression in FNA tumor material from NSCLC patients.
**Fig. S3**. Cytology analyses of FNA tumor material from two different parts of the same tumor lesion.
**Fig. S4**. CT images complementary to Figure 4B.
**Fig. S5**. Functional network analysis related to PD‐L1 signature data.
**Fig. S6**. Functional network analysis related to tumor stage signature data.
**Table S1**. Correlation between immune cell subsets and observed signatures.

## Data Availability

Most data that support the findings of this study are available as Supporting Information. Data not included in Supporting Information are available from the corresponding authors (bo.franzen@ki.se or Kristina.viktorsson@ki.se) upon reasonable request.
