# Supplementary material for: Multiplex immune protein profiling of fine‐needle aspirates from patients with non‐small‐cell lung cancer reveals signatures associated with PD‐L1 expression and tumor stage
Source: Mol Oncol. 2021 May 1;15(11):2941–57. doi: 10.1002/1878-0261.12952 (PMC8564641; doi:10.1002/1878-0261.12952)
Supplement: Supplementary file 1 — Fig. S1. Overview of the study. Fig. S2. Examples of (A) immunocytochemistry (ICC) and (B) immunohistochemistry (IHC) analyses of PD‐L1 expression in FNA tumor material from NSCLC patients. Fig. S3. Cytology analyses of FNA tumor material from two different parts of the same tumor lesion. Fig. S4. CT images complementary to Figure 4B. Fig. S5. Functional network analysis related to PD‐L1 signature data. Fig. S6. Functional network analysis related to tumor stage signature data. Table S1. Correlation between immune cell subsets and observed signatures. [file MOL2-15-2941-s001.pdf]

Supplementary figures and tables

Supplementary figure S1. Overview of the study. (I) NSCLC tumor lesions in the thoracic region were sampled by fine needle aspirations (FNA) during routine examination by CT. (II) The representativity of the material was analyzed on cytology smears with May–Grünwald–Giemsa staining. PD-L1 expression was analyzed by immunocytochemistry (ICC) or by immunohistochemistry (IHC) on FFPE tumor sections. (III) Representative leftover material in the FNA-needle was snap frozen for protein profiling and RIPA extract were made. (IV) The RIPA protein extracts were profiled with the PEA Immune Oncology and Oncology II Multiplex protein assay. (V) Qlucore bioinformatics was used to sort out biomarker signatures. (VI) The clinical and molecular characteristics of the included NSCLC patients (**Table 1**) was used in the protein signature analyses.

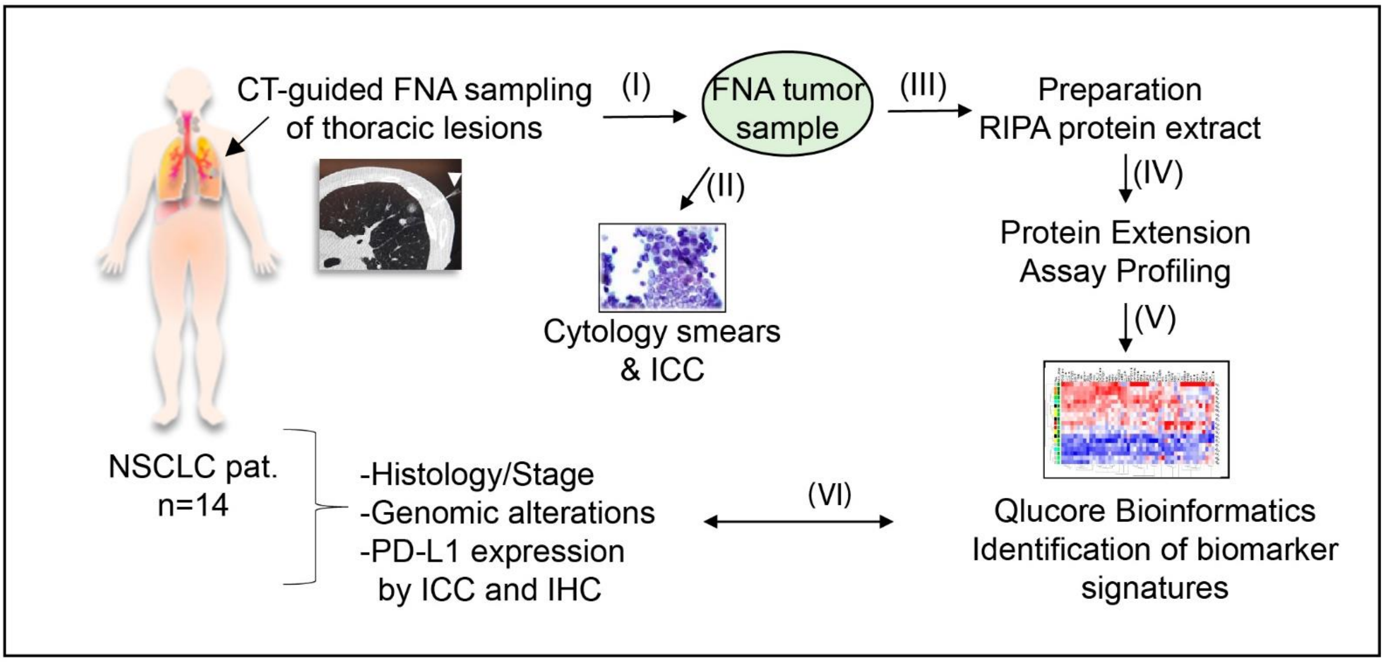

Supplementary figure S2. Examples of (A) immunocytochemistry (ICC) and (B) immunohistochemistry (IHC) analyses of PD-L1 expression in FNA tumor material from NSCLC patients. Images were taken at 20x magnification on an Olympus BX46 microscope. PD-L1 status in these samples were: 40% in Pat.#22, 60% in Pat.#23, 65% in Pat.#3, 30% in Pat.#26 and 5% in Pat.#20.

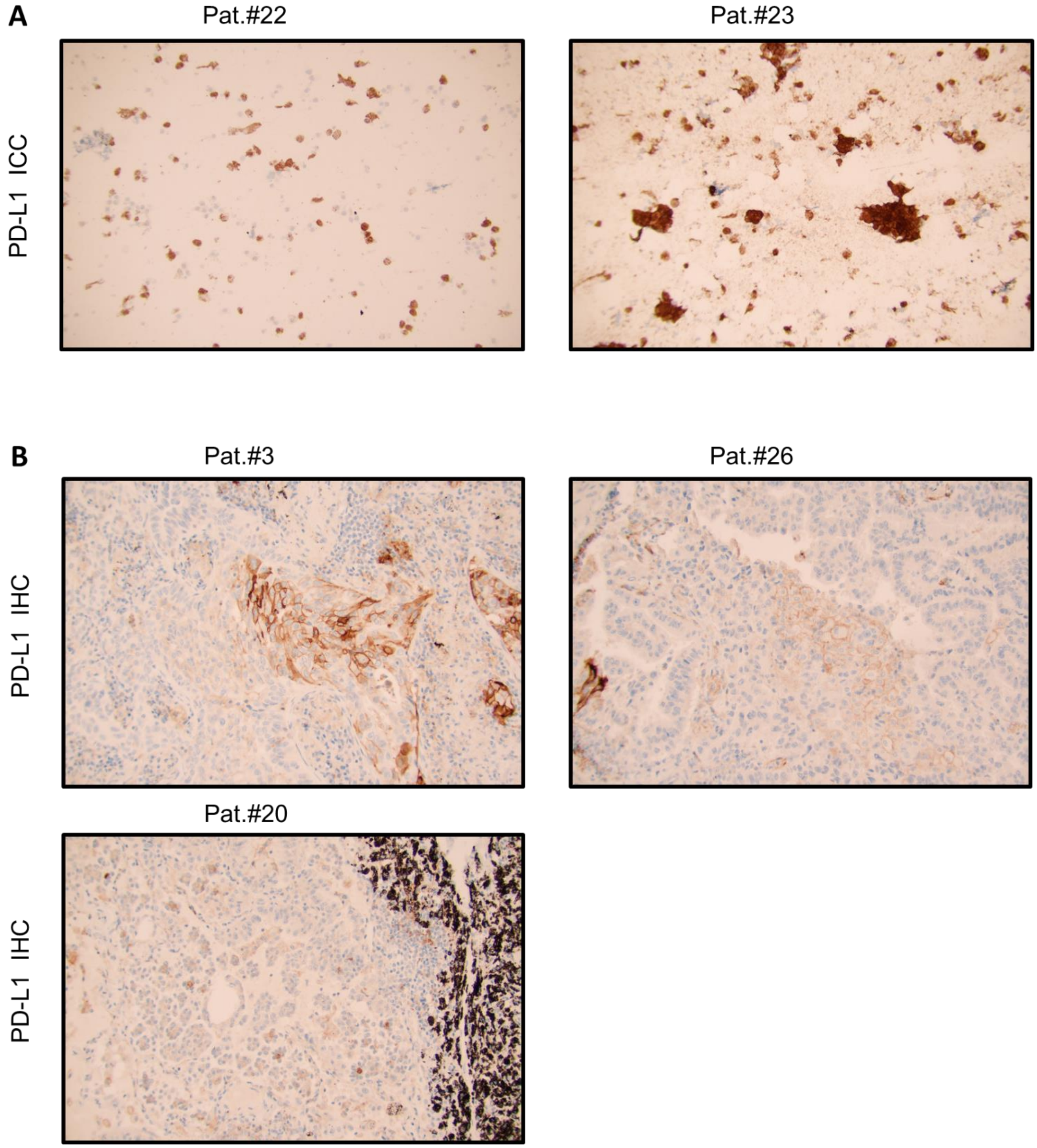

Supplementary figure S3. Cytology analyses of FNA tumor material from two different parts of the same tumor lesion. May–Grünwald–Giemsa staining of smears corresponding to two FNA-samples (A and B) from 6 patients. Images were taken at 20x magnification on an Olympus BX46 microscope. Cropped images are shown. Please note that this figure represents one close up image per slide while for the estimation of relative proportions of various cell types (see **Table 1**), the entire cytology sample *i.e.* all cells smeared across each glass slide were reviewed for each case. In some cases, a higher content of tumor cells was found in the central part of the tumor (*e.g.* pat.#13B) than in periphery (*e.g.* pat.#13A). CT-images form the same patients and sampling sessions are shown in **Supplementary figure S4**.

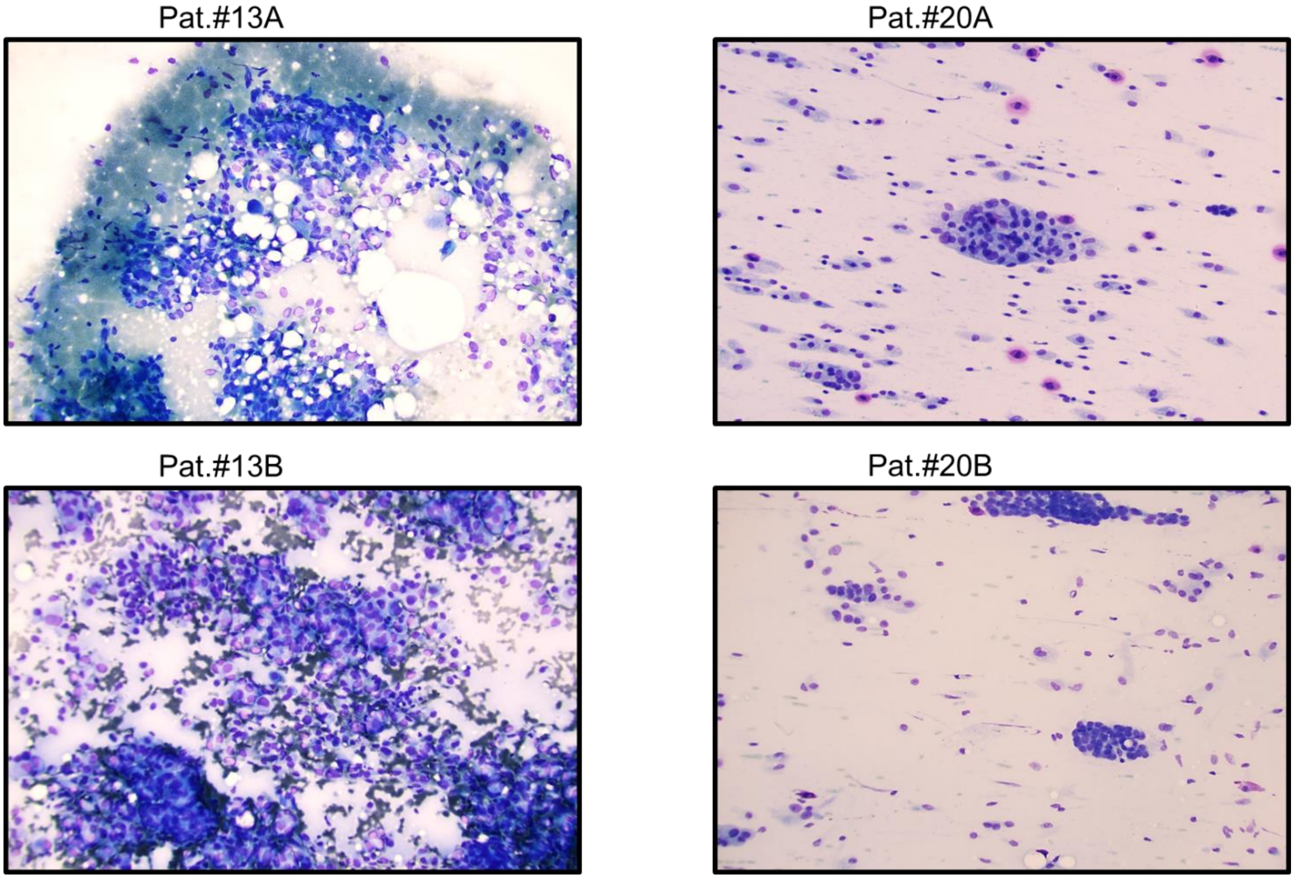

Supplementary figure S3. Cont.

Pat.#22A

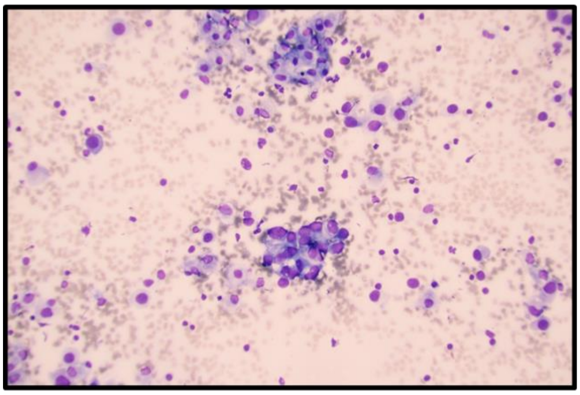

Pat.#24A

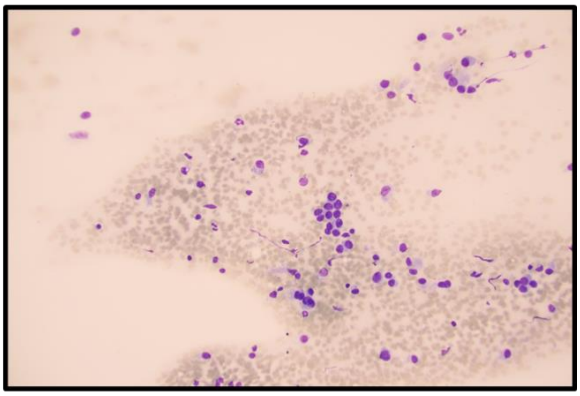

Pat.#22B

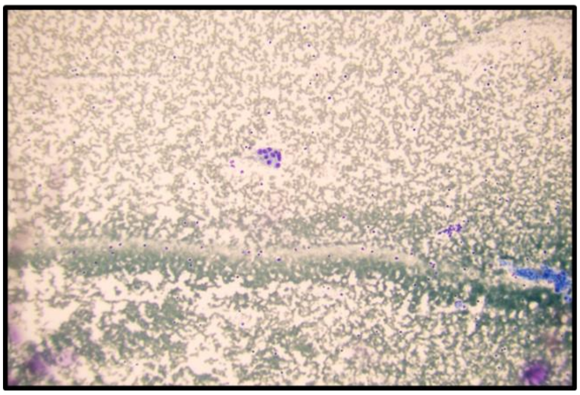

Pat.#24B

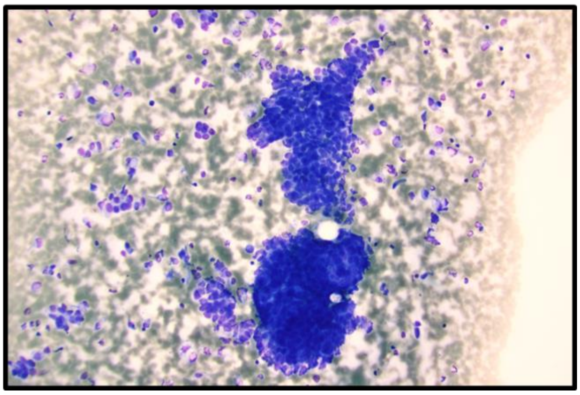

Pat.#26A

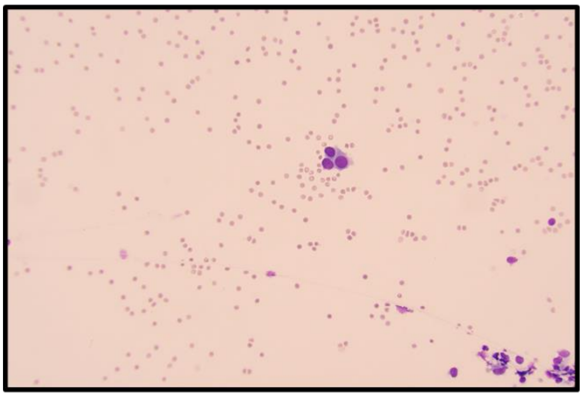

Pat.#27A

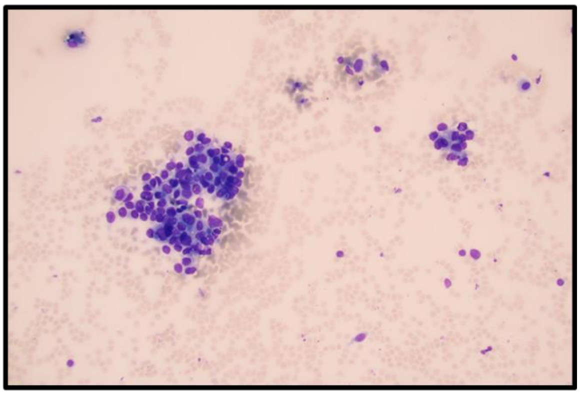

Pat.#26B

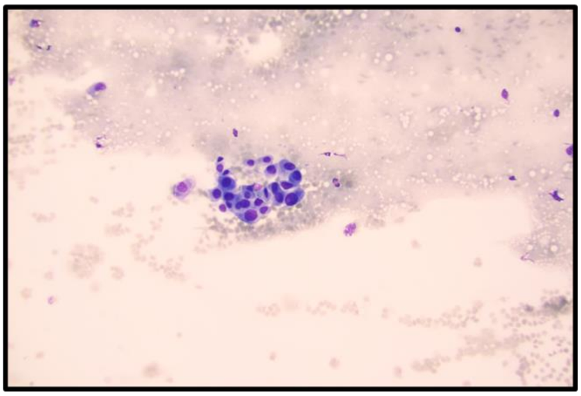

Pat.#27B

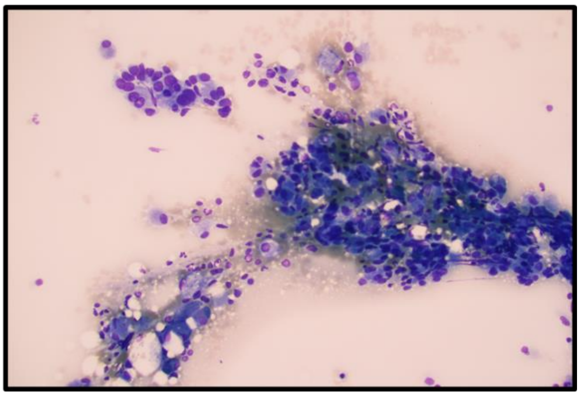

Supplementary figure S4. CT-images complementary to Figure 4B. Additional CT-images captured during the FNA-sampling of patients where two consecutive samples (A and B) were obtained from the same lesion. The FNA-needle can be observed with the tumor marked by the red ring.

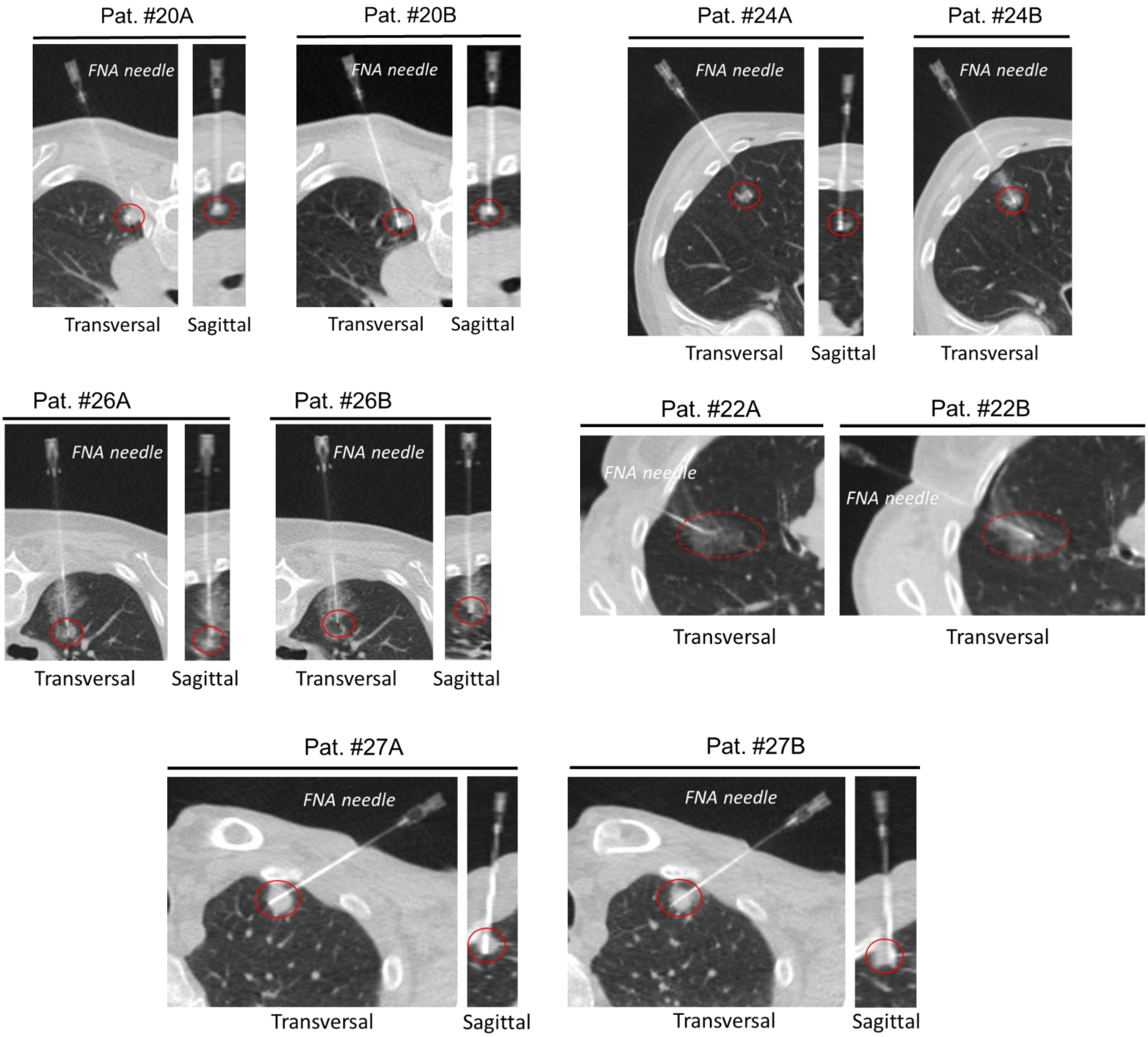

Supplementary figure S5. **Functional network analysis related to PD-L1 signature data.** To further strengthen the PD-L1 associated protein signature data, we implemented a functional network analysis using the String database tool (https://string-db.org) based on the most significant proteins (p=0.01) of the signatures shown in Figure 2 (*i.e.* PD-L1/CD274, CCL3, CCL23, CD83, CD244, CD40, CD5, CD73/NT5E, and EPHA2). Interestingly, this network analysis included automatically CD48 and TNF, proteins that were also part of the signatures revealed by PEA analytics, which would be unlikely if the signatures were random.
Settings: Medium confidence and max number of interactors to show; 1st shell: no more than 10 interactors, 2nd shell: no more than 5 interactors. Colors correspond to biological processes according to the Gene Ontology classification (see legend below). Network statistics: number of nodes: 24, number of edges: 84, average node degree: 7, avg. local clustering coefficient: 0.841, expected number of edges: 23.

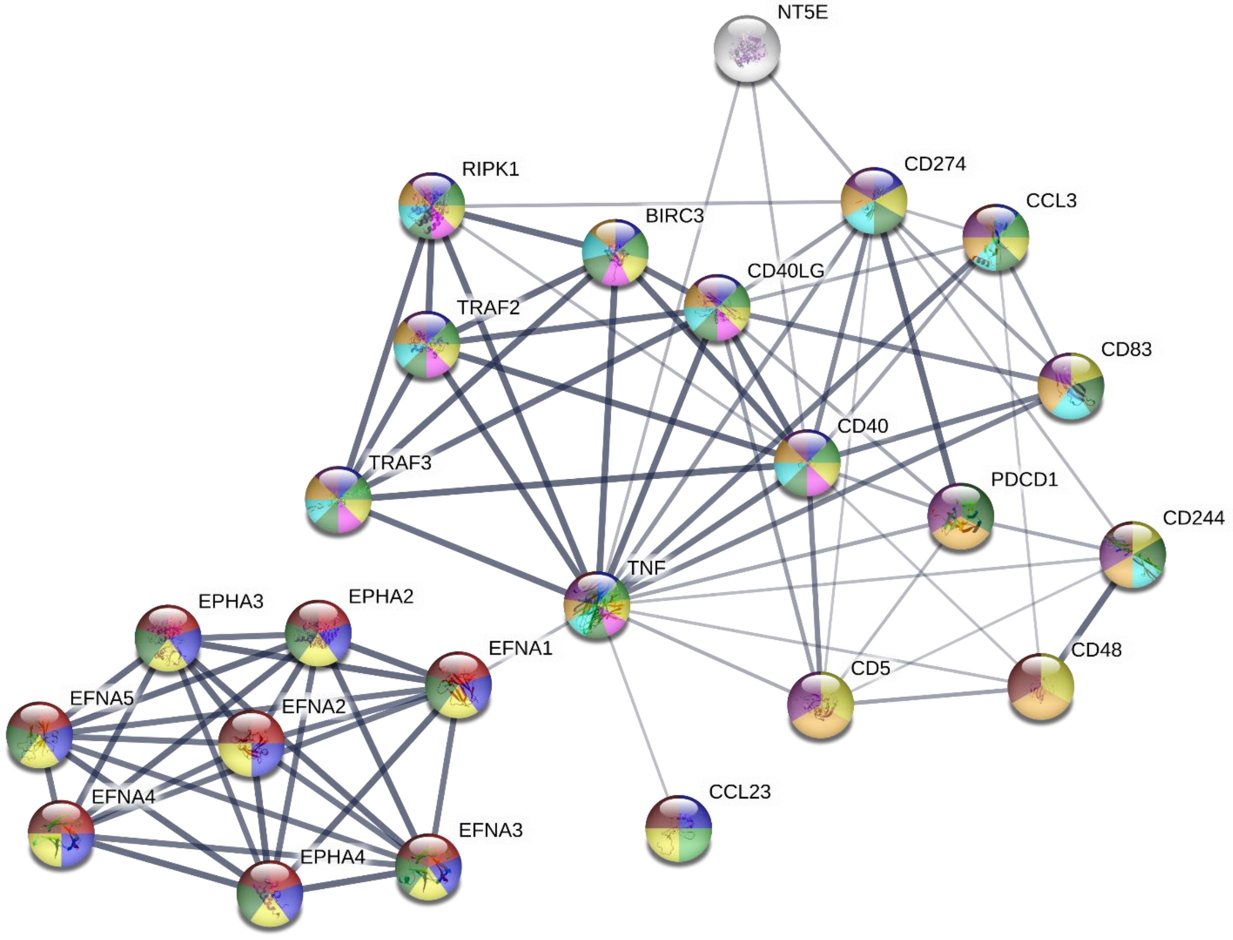

Biological Process (GO)				
GO-term	*description*	*count in gene set*	*false discovery rate*	
GO:0048013	ephrin receptor signaling pathway	8 of 79	1.20e-10	
GO:0007166	cell surface receptor signaling pathway	18 of 2198	4.70e-10	
GO:0071356	cellular response to tumor necrosis factor	9 of 197	7.95e-10	
GO:0007165	signal transduction	22 of 4738	1.51e-09	
GO:0033209	tumor necrosis factor-mediated signaling pathway	7 of 81	2.49e-09	
GO:0051239	regulation of multicellular organismal process	18 of 2788	6.87e-09	
GO:0001817	regulation of cytokine production	11 of 615	8.82e-09	
GO:0002682	regulation of immune system process	14 of 1391	1.28e-08	
GO:0002684	positive regulation of immune system process	12 of 882	1.63e-08	
GO:0040011	locomotion	13 of 1144	1.65e-08	

Supplementary figure S6. Functional network analysis related to tumor stage signature data. To further support the protein signature identified to relate to tumor stage we implemented a functional network analysis using the String database tool (https://string-db.org) based on all 19 proteins (p=0.05) of the signatures shown in Figure 3A. Settings: Medium confidence and max number of interactors to show; 1st shell: no more than 5 interactors, 2nd shell: no more than 5 interactors. Using these setting, 12 of 19 proteins were included in a functional network. To strengthen the statistics according to recommendations from String-DB, settings for 1st and 2nd shell was set to "none": Then, nine proteins remained as a core of the network (labelled with black stars) and the association to "regulation of immune system process" (Gene Ontology) was further improved (PPI enrichment p-value = 5.04e-09). Colors correspond to biological processes according to the Gene Ontology classification (see legend below). Network statistics: number of nodes: 29, number of edges: 66, average node degree: 4.55, avg. local clustering coefficient: 0.559, expected number of edges: 18. PPI enrichment p-value <1.0e-16.

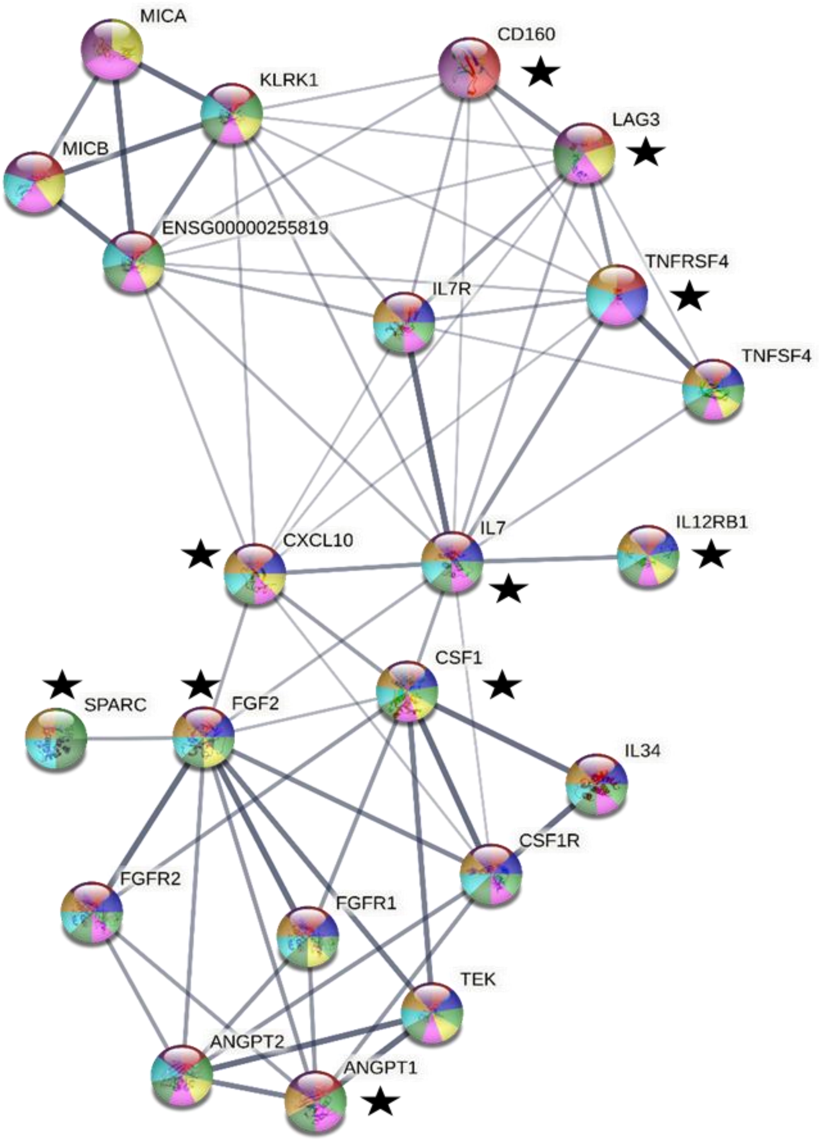

Biological Process (Gene Ontology)

GO-term	description	count in network	strength	false discovery rate
GO:0007166	cell surface receptor signaling pathway	23 of 2198	0.85	6.82e-14
GO:0008284	positive regulation of cell population proliferation	15 of 878	1.06	2.16e-10
GO:0051240	positive regulation of multicellular organismal process	18 of 1551	0.89	2.16e-10
GO:0032101	regulation of response to external stimulus	15 of 955	1.03	4.21e-10
GO:0002376	immune system process	20 of 2370	0.76	5.81e-10
GO:0051239	regulation of multicellular organismal process	21 of 2788	0.71	7.08e-10
GO:0010033	response to organic substance	21 of 2815	0.7	7.34e-10
GO:0042127	regulation of cell population proliferation	17 of 1594	0.86	1.45e-09
GO:0048583	regulation of response to stimulus	23 of 3882	0.6	1.93e-09
GO:0002682	regulation of immune system process	16 of 1391	0.89	2.25e-09

Supplementary table 1. Correlation between immune cell subsets and observed signatures. Data for proteins of interest described in this study (related to Figures 2B, 3A and 4A) showing correlation to immune cell subsets. Data was extracted from the Suppl. tab xlsx-file "NIHMS670442-supplement-2" in Newman et al. 2015. (Newman AM, Liu CL, Green MR, et al. Robust enumeration of cell subsets from tissue expression profiles. Nat Methods. 2015;12(5):453-457. doi:10.1038/nmeth.3337). Arrows represent the observed increase or decrease of a given protein in relation to PD-L1 expression (Fig. 2B, yellow) or stage (Fig. 3A and 4A, blue and green respectively) together with a color in the table matrix representing the subsets of cells where the given protein is expressed above a defined threshold. Several of the shown proteins were previously linked to resistance to immune checkpoint blockade in melanoma, *i.e.* CCL23, CCL13/MCP2, CCL4, CD4, CXCL5, CCL20, GZMA (Chen, P.L. et al. Analysis of Immune Signatures in Longitudinal Tumor Samples Yields Insight into Biomarkers of response and Mechanisms of Resistance to Immune Checkpoint Blockade. Data from Suppl. Tab S8 - Genes sign. Diff. p<0.05).

**Leukocyte subsets & activation states (CIBERSORT, Newman 2015)**	**CCL23**	**CCL4**	**CD244**	**CD4**	**CD40**	**CD5**	**CRTAM**	**IL1A**	**TNFRSF4**	**GZMA**	**CCL13 (MCP4)**	**CD160**	**CSF1**	**CXCL10**	**LAG3**	**IL7**	**MMP12**	**CCL20**	**CD27**	**CXCL5**	**IL12B**	**TCL1A**	**CCL8 (MPC2)**
Correlation to PDL1 (Fig2B)	↗	↗	↗	↗	↗	↗	↗	↗	↗	↗	↗												
Correlation to stage (Fig3A)									↗			↗	↘	↗	↗	↘							
Correlation to stage (Fig4A)									↗			↗	↘	↗	↗		↘	↘	↗	↘	↗	↘	↗
B cells naive																						green	
B cells memory																blue			green				
Plasma cells																			green				
T cells CD8							yellow			yellow					blue				green				
T cells CD4 naive																			green				
T cells CD4 memory resting				yellow						yellow									green				
T cells CD4 memory activated									yellow						blue			green					
T cells follicular helper									yellow						blue				green				
T cells regulatory (Tregs)				yellow		yellow													green				
T cells gamma delta			yellow							yellow		blue			blue								
NK cells resting			yellow							yellow		blue											
NK cells activated		yellow	yellow							yellow													
Macrophages M0													blue							green			
Macrophages M1					yellow									blue	blue								green
Macrophages M2	yellow			yellow							yellow												green
Dendritic cells resting											yellow						green						
Dendritic cells activated									yellow		yellow			blue			green	green			green		green
Mast cells resting																							
Mast cells activated		yellow						yellow										green					
Eosinophils			yellow																				
